# Fungal Degradation of Microplastics—An Environmental Need

**DOI:** 10.3390/toxics14010070

**Published:** 2026-01-12

**Authors:** Rachel R. West, Mason T. MacDonald, Chijioke U. Emenike

**Affiliations:** Department of Plant, Food, and Environmental Sciences, Dalhousie University, Truro, NS B2N 5E3, Canada; rrwest@dal.ca (R.R.W.); mason.macdonald@dal.ca (M.T.M.)

**Keywords:** plastics, polymer, microplastic, fungi, degradation, biodegradation

## Abstract

Plastic waste is a global issue due to the popularity of the product. Over time, plastic degrades into smaller particles known as microplastics and becomes harder to deal with as it easily disperses and can be missed by physical catches. Conventional degradation involves environmental forces like ultraviolet (UV) light, water, temperature, and physical abrasion. However, there is increasing interest in microbial plastic degradation, which could positively impact the transformation of (micro)plastics in various environmental matrices. Most of the available research has focused on bacterial degradation, but there is mounting evidence on the impact of fungal degradation. This review discusses conventional and bacterial degradation, then discusses the advantages of fungal involvement in the degradation of microplastics. Biodegradation enhanced by fungal enzymes is a valuable tool that could greatly improve the removal of these microplastic pollutants from the environment. Due to some biochemical complexities, fungi are naturally omnipresent in marine and terrestrial environments under all sorts of climates. Fungi could thrive by themselves or in association with other microorganisms, which could also be applied in non-biotic plastic degradation processes as an alternative to other forms of plastic management in the environment.

## 1. Introduction

Since its invention in 1839 by Eduard Simon, plastic has steadily gained popularity due to its versatility in numerous applications [1]. With mass production starting in the 1950s at about 1.5 million t [2], the global production reached approximately 368 million t in 2019 [1,3,4]. Hence, it has become pertinent to ask if this could put enormous pressure on the environment. There are numerous chemical combinations to create various forms of polymers, thus ensuring there is plastic to handle any job. Some of the more common or well-known plastics are polyethylene (PE), polypropylene (PP), polystyrene (PS), polyethylene terephthalate (PET), polyurethane (PU), polyamides (PA, Nylon) [2], polyvinyl chloride (PVC), and polyhydroxyalkanoate (PHA) [5]. Biopolymers such as polybutylenesuccinate (PBS), polylactic acid (PLA), and polyhydroxybutyrates (PHAs) are also common [1] and commonly limited to packaging, automotive, and biomedical fields. Biodegradable plastics also exist but are considered less desirable by the industry due to their higher production costs and because some physical properties make them less useful based on the intended purpose, such as plastic mulch [6].

With such overwhelming production of plastic products, waste is inevitable. As with any type of waste, there is a chance for pollutants to enter the environment either from the product itself or from the degradation of the product. Plastic waste comes in various sizes, with larger pieces being known as mega- or macroplastics [7], smaller poly items/particles being given the terms meso- or microplastics [3,4,7,8], and even smaller still known as nanoplastics [1,7]. Mega- or macroplastic waste is easily seen by the human eye and is understood to be an environmental pollutant. Microplastics (MPs) are harder to see and could easily blend in with the environment due to their size (<5 mm), shape, and coloring. Scientific studies have well-documented the prevalence of MPs in all environments, including terrestrial, aquatic, and atmospheric [9]. MPs in aquatic environments tend to be studied the most.

Though most plastic eventually breaks down into smaller particles, approximately only half of the plastic produced is considered biodegradable [2]. Even with its ability to be recyclable or biodegradable, plastic waste can still become a pollutant due to improper handling, disposal, or via accidental release [2,10]. Plastic waste itself can cause entanglement, affixation, and internal biological process blockages, but is not the only concern for pollution. Chemicals used in the production of poly products are also a threat to the environment and human/animal health [11]. There are two main classifications of microplastic waste. Primary MPs are those that directly enter the environment, whereas secondary MPs are particles that have come from the degradation of an original poly item or from the mismanagement of an item containing the poly [8,10]. Many of the poly products produced are considered recyclable.

Microplastics pose a risk to the environment and human/animal health as they are dispersed easily and are difficult to fully capture [8]. The smaller the particles, the greater the chance of them surpassing a capture or removal system [10]. There are various capture methods, ranging from physical to chemical separation, including filtration/sieving, density, magnetic and electrostatic separation, oil extraction, centrifugation, membrane bioreactor, coagulation, and others [1,7,8]. Once the microplastics are separated or collected, they could be broken down through different methods: (1) chemically via the addition of specific chemicals or chemical solutions to break the chemical bonds present in the plastic, (2) physically through the use of impact and various environmental factors (i.e., temperature, light, etc.), which weaken the plastic and cause it to fragment into smaller pieces, and (3) biologically by way of microorganisms using the poly as a food source. Algae [12,13], bacteria, and fungi [1,11] have all been noted as having the potential to aid in the biodegradation of plastic, though fungi are the focus of this review.

## 2. Conventional Degradation of Microplastics

In considering the processes of microplastic degradation, it is important to examine the physical and chemical processes that contribute to their breakdown regardless of biological factors like bacteria and fungi. Microplastic degradation is significantly influenced by environmental forces like ultraviolet (UV) light, water, temperature, and physical abrasion. The photocatalytic reaction of microplastics with UV light triggers the process of photodegradation, which results in the formation of new surface functional groups on the plastic, i.e., hydroxy and carbonyl groups. These modifications affect the chemical reactivity of microplastics and ultimately make them more susceptible to subsequent degradation processes [14,15]. For example, photodegradation is one of the major processes that lead to the fragmentation of microplastics and formation of smaller microplastic particles, potentially down to the level of nanoparticles, which may have altered chemical properties and increased bioavailability in water bodies [16,17].

Physical forces are also significant in the physical breakdown of microplastics. Physical abrasion from wind, hydrodynamic flow, and collisions with other particles is one of the mechanisms that are significantly causing the fragmentation of microplastics into smaller sizes [17,18]. As they occur, microplastics weaken, leading to chain scission—polymer chain fracture—that is accompanied by reduced molecular weight and further fragmentation. This ongoing process of physical degradation is amplified in oceanic conditions where waves and currents consistently apply pressure to plastic waste [19].

Microplastic chemical makeup is also a significant parameter for material degradation. Polymer composition and the incorporation of additives in plastics determine how they respond to environmental stress. For example, polyethylene and polypropylene polymers commonly utilized in microplastic pollution have varying degradation signatures under the same environmental condition. They are reported to degrade at slower rates than biodegradable plastics, thus leading to longer times of microplastic persistence in the environment [20]. The incorporation of specific additives (e.g., colorants, plasticizers, and stabilizers) in these plastics also affects their degradation pathways and the type of their degradation products; these additives generally have the tendency to leach out during degradation, adding additional environmental risks [21,22].

Microplastic degradation also varies with hydrodynamic conditions in aquatic environments, which significantly affects the process of photodegradation. Microplastic degradation by photodegradation depends on the rate governed by water temperature, salinity, and concentration of organic matter because they affect light penetration and microbial activity that may cause further physical disintegration [23]. For instance, elevated temperatures can enhance the degradation rate through increased kinetic energy, while low salinity has been reported to lead to longer microplastic residence times in coastal ecosystems, thereby enhancing their opportunities of contact with marine organisms [24,25].

Microplastic degradation is a multifaceted issue involving an interaction of physical, chemical, and environmental factors. Enhancing the knowledge of microplastic degradation processes is essential to create effective pollution control and mitigation strategies, as well as promoting cleaner surroundings. While numerous studies have been performed on the various impacts of microplastics, additional interdisciplinarity of research must be undertaken to further comprehend the long-term effects of such environmental contaminants, their degradation process, and their broader ecological implications.

## 3. Common Mechanisms of Microbial Biodegradation

Biodegradation is the breakdown of plastics into simpler molecules primarily through enzymatic action of microorganisms [26]. There are four general steps in plastic biodegradation: biodeterioration, biofragmentation, microbial assimilation, and biomineralization [13,27]. Biodeterioration begins once microorganisms establish a presence on the poly waste, and enzymatic secretions then attach and start to penetrate the surface of the polymer [13]. Biofragmentation involves specific chemicals that break down the chemical bonds of the polymer chain into monomers. Assimilation occurs as microorganisms utilize the produced monomers as carbon and energy sources, which leads to further growth. Lastly, the remaining biomass is completely metabolized and biomineralized into carbon dioxide in aerobic environments or methane in anaerobic environments [27].

Major enzymes associated with biodegradation include esterases, cutinases, peroxidases, lipases, proteases, and laccases [28]. Esterases are hydrolase enzymes that split esters into alcohols and acids by the addition of water [28], while cutinases are a subclass of esterases that are categorized by their ability to degrade polymers with a high molecular weight [29]. Peroxidases, also called catalases, catalyze redox reactions to form oxidized and polymerized compounds [30]. Lipases and proteases catalyze the degradation of lipids and long-chain polypetides, respectively [31,32].

Biodegradation can also be enhanced through production of pro-oxidant ions. Pro-oxidant ions improve degradation either by inducing formation of reactive oxidative species or by inhibiting antioxidant systems [28]. Common pro-oxidant ions include Fe^3+^, Mn^2+^, and Co^2+^ [33,34]. Co^2+^, in particular, is a crucial pro-oxidant ion. Even when other ions were present, the presence of Co^2+^ significantly accelerated abiotic plastic degradation [35].

## 4. Bacterial Microplastic Degradation

### 4.1. Impact of Bacterial Species

The role of bacterial communities in the degradation of microplastics is a significant area of study considering the global contamination of microplastics and the resultant environmental hazards. Thus, it is important to discuss the potential of bacterial degradation to plan effective bioremediation strategies and understand a major vector of microbial biodegradation. Bacteria play a crucial role in such degradation through diverse metabolic processes and enzymatic activities to break down microplastic polymers into smaller, less toxic compounds or mineralize them completely.

At present, various bacterial species have been reported to be associated with microplastics, which develop biofilms that enhance their degradation rates. It has been established through experiments that biofilms consisting of multiple bacterial taxa can enhance colonization and, consequently, degradation of microplastics [36]. For example, the presence of various classes of bacteria in association with microplastics, including Tenericutes, Firmicutes, and Deferribacteres, indicates the likelihood of their role in plastic degradation [36]. Gaining knowledge of such microbial communities is indispensable, especially the fact that bacteria not only accelerate degradation processes but also influence the bioavailability of the sequestered pollutants through alteration of the metabolic processes.

Some of the bacteria from the *Pseudomonas* and *Bacillus* genera possess great capacities to accelerate degradation of common plastic forms, i.e., polyethylene and polypropylene. Studies by Hossain et al. [37] found that *Bacillus cereus* showed a degradation rate of about 20% for polyethylene microplastics within 35 days of incubation. Nonetheless, it is noted that other similar studies have documented disparate degradation rates for other microbes, and thus earlier reports might not be in support of the findings consistently; for example, other studies had documented less than 20% degradation rates for some isolates [37].

Marine ecosystems are especially interesting when it comes to microbial degradation of microplastics. Microplastic-residing bacteria are likely to develop specialist adaptations, enabling them to use microplastics as carbon and energy sources. Dudek et al. [38] revealed that bacteria that inhabit microplastics in marine ecosystems include hydrocarbon-degrading bacteria such as *Alteromonas* and *Marinobacter*. In addition to degrading the plastics, these bacteria participate in nutrient cycling in their environments, and thus microbial plastic degradation has double the ecological benefit.

The bacterial enzymatic activities used in the degradation of plastics vary and include diverse enzymes such as hydrolases, laccases, and peroxidases [39]. The enzymes can cleave chemical bonds in polymer matrices, therefore increasing breakdown facilitated by microorganisms. Carr et al. [40], for instance, outlined BgP, a cutinase-like enzyme from marine sponge-derived bacteria that has the capability to degrade polyesters found in microplastics. This highlights that exploring new enzymatic processes can lead to new biotechnological applications for plastic waste disposal.

Moreover, microplastic biofilm formation by microorganisms also contributes to improved efficiency to degradation processes. Biofilms can increase the surface area of the microplastic available for microbial colonization as well as enable better enzymatic interactions [41]. This is significant because microplastics have the tendency to form a biofilm during their exposure to the environment, which influences their degradation processes [42]. Such biofilms developed can also alter the physicochemical characteristics of the microplastics to support or inhibit further microbial attachment and metabolic interactions.

Microplastic–antibiotic resistance interaction is another critical point to consider. Liu et al. [36] explained how microplastics that incorporate some bacterial strains can facilitate the spread of antibiotic resistance genes, thus raising questions about the environmental consequences of microplastic biofilms. This indicates the complex relationship between biodegradation potential and potential environmental health risks induced by microplastic-based biofilms.

In addition, the degradability capacity is not only different among bacterial species but also differs based on the types of plastics involved. Various authors have shown that bacteria are efficient in degrading different types of polymers at different efficiencies. For example, *Rhodococcus* genus strains were found to efficiently degrade polyethylene, implying the importance of proper selection and optimization of microbial strains based on the target plastic type to attain maximum biodegradation productivity [43].

Since microbial degradation of microplastics is influenced by environmental factors (e.g., temperature, pH, nutrient availability), progress in understanding their influence on microbial degradation is crucial to ensure the optimization of bioremediation processes. Optimal conditions for bacterial growth will significantly enhance efficiency in microplastic treatment processes, moving towards ecological sustainability using microbial bioremediation [44].

In general, microbial degradation of microplastics is a new and promising research area with important implications for environmental management. The ability of various bacterial lineages to form biofilms, and their enzymatic capabilities, are central in facilitating the process of degradation. Continued studies on bacteria diversity in microplastic interactions, their metabolisms, and environmental factors governing their activities will be core towards realizing the microbial potential for effective plastic waste remediation.

### 4.2. Challenges of Bacteria-Assisted Microplastic Degradation

Microbial breakdown of microplastics, as one fascinating bioremediation method, has several challenges and limitations. Understanding of the intricacies associated with such limitations is critical in identifying effective ways of managing microplastic pollution in different ecosystems. We touch on below some of the primary challenges faced by bacterial communities in their quest to degrade microplastics.

First and foremost, the heterogeneity of microplastics presents an insurmountable challenge. Microplastics are heterogeneous in composition, shape, size, and surface properties, making the degradation process by microbial communities effectively challenging. The wide variety of polymers requires specific bacterial strains or enzymes to degrade optimally, thus limiting possibilities for bioremediation. For instance, while some bacteria can break down polyethylene, such organisms are not necessarily capable of metabolizing polystyrene, and a varied consortium of microbial populations is needed to address sophisticated microplastic pollution situations [39,45]. This diversity is such that the same technique to biodegrade might be unfeasible.

Additionally, the sophisticated chemical structure of most plastics renders them with high microbial resistance to degradation. Compared to organic compounds, microbial communities that have been adapted to degrade plastics require new enzymes or metabolic pathways to break down [39]. For example, polystyrene and polyethylene are resistant to biodegradation due to the stable carbon–hydrogen bond, making them “forever chemicals” that are difficult to decompose [45,46]. Furthermore, even if some bacteria have enzymes capable of degrading plastics, the enzymes may not function effectively in varied environmental conditions, hindering the decomposition process.

Interactions between plastic and bacterial species within the ecological environment also create challenges. Research indicates that microplastics possess the ability to modify microbial community dynamics, thus impacting the diversity and composition of microbial communities. For example, plastic colonization can favor plastic-degrading bacteria but limit other crucial microbial communities [37,47]. This dysbiosis diminishes the general well-being of the microbial community, limiting synergistic processes contributing to effective degradation. Imbalanced microbial communities also impact nutrient cycling, potentially leading to devastating ecological effects [48].

Biofilm development on microplastics offers possibilities and challenges for microbial degradation. On the one hand, biofilms enhance microbial colonization, and plastic bioavailability can be increased. On the other hand, biofilms might also include pathogenic bacteria or resistant bacterial strains. The process was found in numerous studies, which demonstrated that microplastics can be utilized as reservoirs of harmful microbes [49,50]. Antibiotic resistance genes leached from microplastic-bound bacteria pose further public health issues in the sense that while certain bacterial species can facilitate plastic degradation, at the same time, they can spread pathogenic bacteria.

Environmental factors influencing bacterial activity and microplastic degradation rates also affect efficiency in degradation. Temperature, pH, and the amount of nutrients influence microbial growth significantly [45]. Microbial activity or growth in most environmental settings may not always be beneficial, hence lowering degradation rates of microplastics. For instance, a low temperature can potentially restrain bacterial metabolic processes, lowering plastic degradation significantly [51].

A second issue emanates from competitive interactions within microbial communities. Microplastics may stimulate certain bacterial populations, increasing competition for available nutrients or space within the habitat among diverse microbial communities. Such competition could hamper effective pollutant degradation, thus yielding poor bioremediation [48]. Furthermore, opportunistic pathogen development on microplastics has the potential to take over helpful microbes that can help in the degradation of plastics and therefore reduce the effectiveness of bioremediation processes [36,52].

The accumulation of secondary microplastic fragments, which are the products of the degradation process, is also an issue. Other research suggests that secondary particles, being smaller, can adsorb toxic chemicals or toxic pollutants from the surroundings, posing new risks to organisms that ingest them and entering a cycle of pollution [53]. This is more complicated to study in terms of the ecological impacts of plastic degradation because secondary microplastics have the potential to be even more toxic than the initial substances as they hold aggregated harmful substances.

Practical application of microbial breakdown strategies also faces logistics problems. In situ treatment may be affected by the need for optimal conditions for the desired bacterial species or the need for supplemental nutrients to optimize microbial activity. Such needs may be difficult in natural environments, particularly in aquatic systems where the nutrient content can change dramatically [54]. In addition, bioremediation application in the field with targeted bacteria consortia can prove to be challenging to scale up; having successful microbial blends effectively used across diverse environments is taxing in terms of cost, regulation, and environmental acceptability.

Finally, the duration of microbial degradative processes can be a significant hindrance. In most cases, microbial degradation of microplastics takes decades or even years to yield meaningful results, which poses a threat to the effectiveness and timeliness of such bioremediation measures [24]. The long residence period of microplastics also poses a threat to the viability of solely relying on natural bacterial degradative processes, given the rapid and extensive plastic buildup in environments worldwide.

In all, while the potential for microplastic degradation exists among different species of bacteria, overwhelming factors constrain their effectiveness and efficiency. Variability of the plastic type, biodegradation resistance, ecological interactions among microbial populations, dependency on environmental conditions, competitive interactions, secondary contamination issues, challenges of practical implementation, and the timeframe for degradation all make the challenges to be overcome harder. A multi-strategy tactic combining bacterial degradation with other remedy mechanisms, taking into consideration the environmental context, is essential to respond to the rising threat of microplastic pollution. This includes the need for fungi-assisted degradation.

## 5. Fungal Microplastic Degradation

### 5.1. Impact of Fungal Species

**Figure 1:**
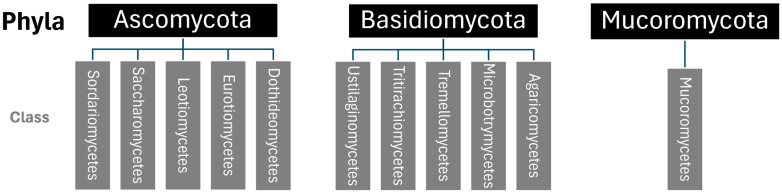
List of major phyla and classes of plastic degrading fungi, created based on the analysis of Ekanayaka et al. [55].

There are an estimated 2–4 million fungal species in existence, but only about 100,000 have been documented and grouped into 19 main fungal phyla [2]. Of these 19 phyla, plastic-degrading fungi tend to belong to one of three fungal phyla: Ascomycota, Basidiomycota, and Mucoromycota [55]. Most-plastic degrading fungi belong to Ascomycota [55]. Major phyla and classes of plastic-degrading fungi are shown in Figure 1.

Fungi can survive as “free-living” organisms known as saprotrophs or be found in “mutualistic associations” that can be symbiotic (i.e., mycorrhizas) or parasitic [2]. Fungi are present everywhere. Due to the sheer number and variety of species, there are fungi found ubiquitously throughout all environments and all levels of those environments [2]. They display various abilities to degrade substrates regardless of environmental limitations, such as low moisture, low nutrient concentrations, and acidic pH [56]. Fungi have different morphologies based on the environment and the needs of microorganisms. The mostly commonly used morphologies include unicellular and dimorphic types [57], with filamentous morphologies being commonly mentioned in poly biodegradation studies [2].

Fungi attach themselves to a plastic film and then break down their food sources through the process of enzyme excretion. Much like bacteria, these enzymes target the chemical bonds present in a compound, breaking them down [2] or oxidizing them to become non-toxic or less toxic compounds [56]. Enzymes such as manganese peroxidase, lignin peroxidase, versatile peroxidase, laccase [13], depolymerase [58], esterases, lipases, proteases, and ureases [59] are all shown to degrade various types of plastic polymers. Similarly, some fungal species have been known to excrete siderophores, which can attract some metals, thus aiding in the removal of metal toxicity [2]. Many plastics are petroleum-based, containing hydrocarbon monomers or other types of carbon monomers. The fungal biodegradation of these hydrocarbons is usually completed aerobically but has also been documented by filamentous fungi to happen anaerobically [56]. The ease with which a material can be biodegraded is based on the compounds found within the material and the types of bonds within that compound [56] as well as the environment in which the material has been deposited.

Many plastics are responsive to biodegradation via fungal means in both terrestrial and marine environments (Table 1). Zhou et al. [5] found success in biodegrading PE, PVC, and PHA in bovine manure. Although the resulting compost was of lower quality than that of compost not containing microplastic with decreased fungal biodiversity, the plastic was still completely biodegraded. Ali et al. [13] reviewed a few studies that found biodegradation of PSPU (polyester polyurethane), PU-acrylate copolymer, PE, PP, PS, and PCL (polycaprolactone) to be possible, although these studies also showed that the use of a consortium of fungal and bacterial microbes was better prepared to handle the breakdown when compared to a single microorganism. Radzi et al. [60] also discussed the use of microbial consortiums or communities to enhance and increase the efficiency of the biodegradation process of PAH-plastics.

Fungal degradation studies follow similar procedures when it comes to studying the biodegradation potential of plastic waste. They identify the plastics or the environment with the plastics issue, sample the environment(s), plate the samples, isolate the cultures that grow, identify the organisms in the culture, multiply the isolated organism and test on the poly under a controlled environment, assess, and test in the “field”. The source of the microbes to be used is considered very important [60], but not much attention has been paid to the season or timing of the sampling. It would be important to understand which fungal cultures (or other microbes) operate during the seasons of a given environment.

### 5.2. Fungal Species Biodegradation Modes of Action

#### 5.2.1. Activation of Pentose Phosphate Pathway (PPP)

The type and composition of carbon substrate digested by fungi will affect carbon metabolism [76]. Many synthetic polymers resist biodegradation, which is one of several reasons plastics are so useful. But by resisting biodegradation, plastics impose a form of carbon starvation on fungi [77]. Fungi will often activate the oxidative PPP as a survival mechanism during times of carbon starvation, where glucose-6-phosphate is converted to ribulose-5-phosphate via three steps while producing the reduced form of nicotinamide adenine dinucleotide phosphate (NADPH) [78]. The ribulose-5-phosphate is then available for the non-oxidative PPP pathway [78].

The PPP serves several immediate purposes: (1) it increases the reducing power of the cell through NADPH production, (2) it produces ribulose-5-phosphate as a precursor to nucleotide synthesis, and (3) it feeds into the non-oxidative PPP to produce erythrose-4-phosphate as a precursor to aromatic amino acid synthesis [78]. With respect to plastic degradation in fungi, activation of the PPP also produces glycoside hydrolases and oxidoreductases [77]. These enzymes are adapted to degrade lignocellulose in plant tissue and several of them can degrade plastic [77].

#### 5.2.2. Penetration of Fungal Hyphae

A high molecular weight, hydrophobicity, and insolubility are other challenges with plastic biodegradation by fungi [79]. Filamentous fungi can mitigate these challenges. Most fungi are filamentous [80], including many species of Ascomycota, Basidiomycota, and Mucoromycota [81]. Filamentous fungi have a unique vegetative structure consisting of a rigid, tubular hyphae that can be extended into a variety of materials [56,82].

Hyphae have a lot of senses that can respond to a variety of environmental or physical factors [83]. Plastics have hydrophobic surfaces, and fungal cells respond to this stimulus by producing HsbA proteins [84]. HsbA proteins fuse to the catalytic domains of hydrolytic enzymes, improving the adsorption characteristics of the enzymes and permitting them to attach to plastic surfaces [85]. Such enzymes break down carbon chains and weaken plastic polymers by creating pits or cracks. As the hyphae penetrate the weakened cracks, they can augment the degradation process by applying mechanical pressure [86].

#### 5.2.3. Extracellular and Intracellular Enzymatic Biodegradation

Success of fungal-induced plastic biodegradation is often dependent on the type of parent plastic, which are polyolefin or non-polyolefin. Polyolefins, such as PE, PS, and PP, are highly resistant to microbial attack. Fungi must excrete strong extracellular enzymes such as laccase, manganese peroxidase, lignin peroxidase, oxygenases, and hydroxlases in order to gradually erode polyolefin surfaces [2]. A combined effort from enzymes and physical degradation, such as UV or ozone, greatly accelerates initial degradation [87].

The diversity of fungal enzymes allows for effective degradation of many plastics through multiple modes of action (Table 2). The majority of plastic-degrading fungal enzymes are classified as either hydrolases or oxidoreductases [88]. Of the hydrolases, cutinase, esterase, lipase, and urease will degrade PUR, and cutinase, lipase, and polyesterase will degrade PET [77,88]. Oxidoreductases, including hydroxylase, laccase, oxygenase, and peroxidase, are effective at degrading PE [77,88]. There is comparably less work on enzymatic degradation of plastics like PS and PP. However, Srikanth et al. [28] have written a review describing multiple fungal species that have been effective in degrading PP and PS, though information on the mode of action was limited.

**Figure 2:**
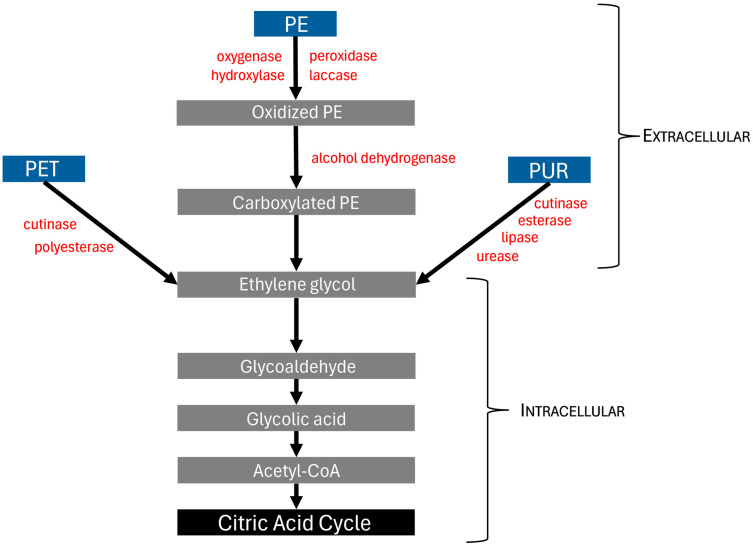
Pathway and extracellular enzyme involvement in catabolism of three major plastics to enter the citric acid cycle. The figure represents an abbreviated pathway as described by Okal et al. [77]. PE: polyethylene; PET: polyethylene terephthalate; PUR: polyurethane.

As biodegradation continues, microplastic molecules may be absorbed into the cell, where they are further reduced. Intracellular enzymes, such as oxidoreductases, create products like glycerate and glyoxylic, butyric, acetic, glycolic, caproic, and carboxylic acids [89]. These metabolites are eventually converted into acetyl-CoA to enter the citric acid cycle via acetyl-CoA C-acyltransferase [90]. Although the citric acid cycle is thought to be the final destination in the breakdown of most microplastics, there is evidence that some microplastics enter the PPP or glyoxylate shunt pathway [91,92]. An abbreviated pathway of extra- and intracellular plastic catabolism is shown in Figure 2.

### 5.3. Advantages of Fungal Biodegradation

Much of the current research on microbial plastic biodegradation focuses on bacteria [93]. Bacteria are effective biodegrading organisms and are covered in detail in multiple reviews [94,95,96]. However, fungi offer several advantages over bacteria that make them excellent candidates for microplastic biodegradation. For example,

(a) Fungi are extremely adaptable to extreme and diverse environmental conditions, such as those whose pH, temperature, moisture, or other environmental parameters may not be suitable for bacteria. For example, some *Aspergillus* and *Penicillium* species can survive in environments with a pH ranging from 2 to 11 [97]. A study testing 14 fungal species found that all except 1 could tolerate at least 40 °C at high humidity, and 9 species tolerated at least 50 °C [98]. Under dry conditions, all fungi tolerated 70 °C while several tolerated more than 100 °C [98].
(b) Fungi have a stronger plastic affinity than bacteria, which can be attributed to the involvement of fungal-specific hydrophobins. Such hydrophobins help attach hyphae to plastic surfaces [99]. As noted earlier, hyphae can also produce HsbA proteins to improve absorptive characteristics of enzymes as well [84].
(c) Fungi have a diverse range of highly efficient enzymes. The hydrolase found in fungal extracellular enzymes is effective at hydroxylation of long-chain compounds, while multiple enzymes in the oxidoreductase system have plastic-degrading capabilities [89,100]. For example, white rot fungi (*Phanerochaete chrysosporium*) produce a host of ligninolytic enzymes that have demonstrated plastic-degrading properties [93,101].
(d) Fungi form symbiotic relationships with plants. More than 80% of land plants partner with fungi to assist with nutrient extraction through mycorrhizal associations [102,103]. Although many biodegradation studies have been conducted in laboratory settings, there is the additional possible benefit to plants and biodiversity when biodegradation happens in nature. Symbiosis also enhances the overall effectiveness of fungal biodegradation as it allows for the expansion of fungi through the root network of plants, potentially accessing additional microplastics [104].
(e) Fungi use mechanical force to help degrade plastics. As noted above, hydrolytic extracellular enzymes weaken plastic and form cracks or pits, through which the hyphae can apply mechanical force [86]. Wu et al. [93] noted significant pitting and erosion from white rot fungi on plastic that then resulted in fragmentation.
(f) Fungi offer superior versatility for microplastic biodegradation. Fungi microplastic biodegradation is effective in water and on land, as well as in situ and ex situ [104].
(g) Fungi have a higher carbon use efficiency and release less CO_2_. Carbon use efficiency is 40–55% for fungi compared to only 20–30% for bacteria [105]. This was further confirmed by researchers who heated sterile soil inoculated with either bacteria or fungi and found the fungal soil released less CO_2_ [106]. It is suggested that fungi are able to produce enzymes capable of building more stable organic building blocks [106].

### 5.4. Limitations to Fungal Biodegradation

Fungal biodegradation is a positive progressive method to aid in the remediation of poly pollution. However, fungal bioremediation comes with its limitations. Biodegradation is known to take time and is greatly influenced by the environment and the substrate involved in the process [1,11]. Environmental conditions, such as temperature, exposure to light, moisture, and others, can be both positive and negative factors when analyzing sample/particle degradation time [11]. Anik et al. [7] mentioned the negative implications of the inability to control environmental factors. The season, region, and lack of predictability cause variances in environmental conditions that not only inhibit the microbes but also make experimental replication difficult. Zhou et al. [5] identified different carbon sources (i.e., microplastic and initial compost) favoring certain microorganisms, resulting in a loss of overall diversity in the fungal community. The chemical structure of plastic makes some types of plastics more difficult to degrade than others [1,13], and the potential release of toxic substances during the degradation process can also limit microbial progression [27].

The best methods to assess fungal success in biodegradation are under debate [2,7]. Due to the microscopic size, biofilm development, cost of some analyses, and the environment in which the breakdown of the substrate happens, it can be difficult to prove that fungi are actively degrading plastic waste or whether degradation just happens to occur in the presence of fungi. Zeghal et al. [2] emphasized various methods of quantifying the mass loss of plastics or growth of fungal colonies while on plastics, which all have their drawbacks and shortcomings of proving without a doubt that the plastic is becoming smaller only due to the fungus using it as the food source. Sang et al. [107] support this by stating how difficult it is to measure fungal biomass growth involving complex matrices. Finally, gaps in knowledge of fungi, their species (especially marine), identification(s), and their processes of full fungal biodegradation remain another considerable limitation [2].

## 6. Conclusions

Biodegradation is a very important, complex, and vital process needed to help retake the environment from the threat of microplastics. Although not the only option, fungi are an extremely credible option due to the variety of species, abilities, and omnipresent existence. Understanding the limitations of fungal activities and the ideal environment in which to exhibit biodegradative abilities is key to ensuring optimal efficiency in combating the plastic pollution.

It is evident that more work and research need to go into enabling the creation of more infrastructure to support the biodegradation process, whether this be through space, material handling, or the replication of the desired environmental conditions to either reproduce the fungal colonies or to complete the biodegrading processes. Much evidence supports the amalgamation of microbial groupings (especially fungi, bacteria, algae) working together as the most efficient way to handle biodegradation due to limited capacities of individual microbes or microorganisms [13]. The addition of a catalyst-type factor or pre-treatment, such as an improved desired environmental factor or addition of fungi/microorganism-safe chemical to aid in a part of the process, might also increase efficiency or the speed of biodegradation.

Future studies need to continue to investigate all aspects of the role fungi play in the biodegradation of plastics, with specific attention being granted to creating optimal conditions to improve the efficiency of these microbes. Certain knowledge gaps require attention—ratios of optimum food source versus amounts or types of fungi, the identification of more species of fungi to attach the various types of plastics as well as the various environments, and/or finding the various stages where the fungi (or other microorganisms) lag and could be artificially energized. Consideration also needs to be put into changing the mindset of consumers—so more policymakers and people are coming on board with alternatives to traditional plastic products. However, no matter the outcome of any individual research study, it will take collaboration and cooperation between multidisciplinary fields to combat the global microplastic pollution problem.

## Figures and Tables

**Table 1:** Fungal species with evidence of microplastic degradation. The degradation rate was calculated from the average mass reduction divided by the number of incubation days in each study. PE = polyethylene, LDPE = low-density polyethylene, PET = polyethylene terephthalate, PUR = polyurethane, PS = polystyrene, PVC = polyvinyl chloride.

Species	Plastic	Source	Degradation Rate (%/day)	Reference
*Alternaria alternata*	PE	Coastal plastic	n/a	[61]
*Alternaria* sp.	LDPE	Landfill	0.33	[62]
*Aspergillis tubingensis*	PUR	Soil	1.50	[63]
*Aspergillus flavus*	PE	Seawater	0.54	[64]
*Aspergillus flavus*	LDPE	Soil	1.07	[65]
*Aspergillus fumigatus*	PE	Seawater	0.68	[64]
*Aspergillus glaucus*	PE	Mangrove soil	0.95	[66]
*Aspergillus japonicas*	LDPE	Soil	1.29	[65]
*Aspergillus niger*	PE	Seawater	0.65	[64]
*Aspergillus niger*	PE	Mangrove soil	0.58	[66]
*Aspergillus niger*	PE	Laboratory	0.53	[67]
*Aspergillus niger*	PET	Laboratory	0.10	[67]
*Aspergillus niger*	PS	Laboratory	0.13	[67]
*Aspergillus nomius*	LDPE	Soil	0.15	[68]
*Aspergillus sclerotiorum*	PE	Laboratory	0.20	[67]
*Aspergillus sclerotiorum*	PET	Laboratory	0.15	[67]
*Aspergillus sclerotiorum*	PS	Laboratory	0.27	[67]
*Aspergillus sydowii*	PE	Mangrove dumpsite	0.63	[69]
*Aspergillus tereus*	PE	Mangrove dumpsite	0.70	[69]
*Aspergillus tereus*	PE	Seawater	0.73	[64]
*Auricularia auricula*	PS	Laboratory	0.11	[70]
*Candida albicans*	PE	Laboratory	0.17	[67]
*Candida albicans*	PET	Laboratory	0.07	[67]
*Candida albicans*	PS	Laboratory	0.07	[67]
*Fusarium* sp.	LDPE	Soil	1.14	[65]
*Ganoderma lucidum*	PS	Laboratory	0.05	[70]
*Gloeophyllum trabeum*	PS	Laboratory	2.50	[71]
*Mucor* sp.	LDPE	Soil	0.71	[65]
*Penicillium simplicissimum*	PE	Soil	0.42	[72]
*Penicillium* sp.	PE	Seawater	1.45	[64]
*Penicillium* sp.	LDPE	Soil	0.71	[65]
*Pestalotiopsis microspora*	PUR	Plant stems	n/a	[59]
*Phanerocheate chrysosporium*	PVC	Laboratory	1.11	[73]
*Pleurotus cornucopiae*	PS	Laboratory	0.11	[70]
*Pleurotus ostreatus*	PS	Laboratory	0.32	[70]
*Pleurotus pulmonarius*	PS	Laboratory	0.15	[70]
*Pleurotus sajor caju*	PET	Laboratory	1.45	[74]
*Trametes* sp.	LDPE	Landfill	0.32	[62]
*Trametes versicolor*	PET	Laboratory	0.06	[74]
*Zalerion maritimum*	PE	Laboratory	2.03	[75]

**Table 2:** List of fungal plastic degrading enzymes, their classification, and target plastics. This table was created based on work from Okal et al. [77] and Temporiti et al. [88]. PE: polyethylene; PET: polyethylene terephthalate; PUR: polyurethane.

Enzyme	Enzyme Class	Function	Plastics Degraded
Cutinases	Hydrolase	Cleaves cutin	PUR, PET
Esterases	Hydrolase	Hydrolyzes ester bonds	PUR
Hydroxylases	Oxidoreductase	Adds hydroxyl group	PE
Laccases	Oxidoreductase	Oxidizes aromatic compounds	PE
Lipases	Hydrolase	Hydrolyzes ester bonds	PUR, PET
Oxygenases	Oxidoreductase	Oxidizes aromatic compounds	PE
Peroxidases	Oxidoreductase	Oxidizes various substrates	PE
Polyesterases	Hydrolase	Hydrolyzes PET	PET
Urease	Hydrolase	Hydrolysis of urea	PUR

## Data Availability

No new data were created or analyzed in this study. Data sharing is not applicable to this article.
